# Zhaqu compound improves glucose and lipid metabolism in T2DM with MASLD by modulating gut microbiota and PPARγ

**DOI:** 10.3389/fnut.2026.1775686

**Published:** 2026-03-27

**Authors:** Yifan Liu, Yizhu Xie, Wenjing Yang, Yuchi He, Jialong Jia, Sihan Peng, Xiyu Zhang, Ya Liu

**Affiliations:** 1 Department of Endocrinology, Hospital of Chengdu University of Traditional Chinese Medicine, Chengdu, China; 2 Regulating Metabolic Diseases Key Laboratory of Sichuan Province, Hospital of Chengdu University of Traditional Chinese Medicine, Chengdu, China

**Keywords:** gut microbiota, metabolic syndrome-associated steatohepatitis (MASLD), PPARγ, type 2 diabetes mellitus (T2DM), Zhaqu compound (ZQC)

## Abstract

**Introduction:**

Zhaqu Compound (ZQC), a traditional Chinese herbal formula, has shown promise in improving glucose and lipid metabolism, but its mechanisms in T2DM-MASLD remain unclear.

**Methods:**

In this study, db/db mice with T2DM-MASLD were treated for 12 weeks with ZQC, metformin, or saline. Metabolic outcomes included blood glucose, insulin resistance indices, lipid profiles, hepatic steatosis, hepatocyte ultrastructure, gut microbiota (16S rRNA), fecal metabolomics, and liver transcriptomics. In high-glucose + palmitate–induced HepG2 cells, ZQC-containing serum and the PPARγ agonist GW1929 were applied to assess gluconeogenic and lipogenic proteins.

**Results:**

ZQC improved glycemia, insulin resistance, lipid profiles, and hepatic steatosis. Gut microbiota analysis showed modulation of key metabolic genera, including *Bacteroides, Mucispirillum, Lactobacillus, Lachnospiraceae*, and *Muribaculaceae*, with correlations to glucose, lipid, and hepatic biochemical indices. Metabolomics and transcriptomics revealed regulation of amino acid metabolism, AGE-RAGE/mTOR pathways, and enrichment of hepatic PPARγ–related pathways, alongside modulation of fatty acid and arachidonic acid metabolism. In HepG2 cells, ZQC reduced lipid accumulation, decreased triglycerides and cholesterol, enhanced glucose uptake, and restrained PPARγ and its downstream transporters CD36 and FABP4. N-phenethylhexadecanamide, palmitic acid, and isoxanthohumol likely mediate these effects.

**Discussion:**

Overall, ZQC improves glucose and lipid metabolism in T2DM with MASLD by modulating gut microbiota, influencing hepatic PPARγ–related pathways, and enhancing hepatocyte metabolic function.

## 1 Introduction

Type 2 diabetes mellitus (T2DM) and metabolic dysfunction-associated steatohepatitis (MASLD) are closely linked chronic metabolic disorders that often coexist and exacerbate each other, forming a vicious cycle of glucose and lipid dysregulation. MASLD is now the leading cause of chronic liver disease worldwide, with a prevalence of about 32.4%, and its prevalence in individuals with T2DM can reach 65% (1, 2). Persistent hyperglycemia increases hepatic glucose burden and promotes abnormal lipid accumulation, thereby accelerating the progression of MASLD toward steatohepatitis, liver fibrosis, and even hepatocellular carcinoma (3, 4). Additionally, patients with T2DM who develop MASLD face elevated risks of systemic complications, including retinal disease, chronic kidney disease, peripheral neuropathy, and cerebrovascular events (1, 2). These findings highlight an urgent need for therapies that can address both conditions, yet no ideal agents currently achieve this. Clarifying the pathological basis of their coexistence is therefore critical for advancing therapeutic development.

The gut microbiota is a complex intestinal microbial community that acts as a key interface linking diet, metabolic regulation, and inflammation. It plays an essential role in the development and progression of T2DM complicated by MASLD (5, 6). Under physiological conditions, the gut microbiota regulates hepatic glucose and lipid metabolism by maintaining microbial balance, producing metabolites that modulate lipid metabolism, and preserving immune homeostasis (7, 8). Therefore, modulation of the gut microbiota has emerged as a key strategy for managing MASLD in patients with T2DM. Notably, peroxisome proliferator-activated receptor gamma (PPARγ) plays a central role in regulating lipid metabolism and insulin sensitivity and has been shown to closely interact with gut microbiota function (9, 10). This receptor may serve as a crucial molecular target mediating gut–liver communication and linking microbial signals to hepatic metabolic regulation.

In recent years, traditional Chinese medicine (TCM) has shown considerable potential in the treatment of T2DM complicated by MASLD (11–13). Zhaqu Compound (ZQC) consists of eight Chinese herbal ingredients, including *Crataegus* spp., *Astragalus membranaceus*, Medicated Leaven, *Atractylodes macrocephala*, and *Fructus Aurantii Immaturus*, among others. Preliminary studies indicate that ZQC exerts lipid regulating effects and reduces hepatic lipid accumulation (14–16). Several of its herbal components have been reported to alleviate MASLD and T2DM by modulating the gut microbiota and its metabolites, thereby improving hepatic glucose and lipid metabolism and reducing lipid deposition (17–19). However, whether and by what mechanisms ZQC achieves its overall hypoglycemic and hypolipidemic effects through modulation of the gut microbiota and related metabolic pathways remains to be systematically investigated.

Therefore, this study aims to systematically evaluate the therapeutic effects of ZQC on T2DM complicated by MASLD using an integrated approach combining network pharmacology, multi-omics analyses, and *in vitro* and *in vivo* experiments. Mechanistic investigations will focus on the gut microbiota, microbial metabolites, and key signaling pathways.

## 2 Materials and methods

### 2.1 Reagents

The reagents and antibodies used were the Total Protein (TP) Assay Kit (1,000 tests, P0006, Biyuntian Biotechnology, China), Glucose Assay Kit (96 T, A154-1-1, Nanjing Jiancheng Bioengineering Institute, China), Total Cholesterol (T-CHO) Assay Kit (96 T, A111-1-1, Nanjing Jiancheng Bioengineering Institute, China), Triglyceride (TG) Assay Kit (96 T, A110-1-1, Nanjing Jiancheng Bioengineering Institute, China), Sequencing Reagent Kit (NovaSeq 6,000 SP Reagent Kit V1.5, Illumina, USA), RNA Mini Kit (Qiagen, Germany), DMEM High Glucose Medium (PM150210, Procell, China), Trypsin (S310JV, Shanghai Yuanpei, China), Fetal Bovine Serum (C04001-500, Vivacell, China), Double Antibody (S110JV, Shanghai Yuanpei, China), PPARγ agonist (HY-146480, MCE, USA), Palmitic acid (H8780, Solarbio, China), Oil Red O staining kit (G1262, Solarbio, China), 2-NBDG fluorescent probe (HY-116215, MCE, USA), BCA protein concentration assay kit (BL521C, Biosharp, China), SDS-PAGE Protein Loading Buffer (5×) (BL502A, Biosharp, China), ECL Chemiluminescent Substrate (BL520B, Biosharp, China), Western Blot & IP Cell Lysis Buffer (P0013, Beyotime, China), PBS Buffer (PB180327, Procell, China), and the primary antibodies *β*-Microtubulin (AC026, Abclonal, China), PEPCK (ET7107-29, Huabio, China), G6Pase (A21168, Abclonal, China), GLUT2 (A12307, Abclonal, China), SREBP-1C (ER1917-19, Huabio, China), ACC1 (ET1609-77, Huabio, China), FASN (R1706-8, Huabio, China), PPARγ (A19676, Abclonal, China), CD36 (ET1701-24, Huabio, China), and FABP4 (ET1703-98, Huabio, China).

### 2.2 Preparation of ZQC

Zhaqu Compound (ZQC) is a granule formulation prepared as a water extract from a mixture of eight herbs: *Crataegus pinnatifida* (30 g), *Astragalus membranaceus* (30 g), *Mass Medicata Fermentata* (20 g), *Atractylodes macrocephal*a (15 g) and four additional herbal components. All herbal ingredients were authenticated by certified medicinal plant specialists, and granule preparation was completed at the Affiliated Hospital of Chengdu University of Traditional Chinese Medicine. The adult daily clinical dose corresponds to 46 g of crude drug. Following the methodology outlined in Pharmacological Methods of Traditional Chinese Medicine (Chen, 2011), the mouse equivalent dose was calculated using a body surface area-based conversion formula: Mouse dose (g/kg) = (Adult daily dose/70 kg) × 7.55, where 7.55 is the conversion factor derived from the body surface area ratio between a 70 kg adult and a 40 g mouse. The mouse doses for ZQC and the positive control drug, metformin hydrochloride sustained-release tablets (Glucophage, National Drug Approval No. H20023370), were calculated based on their respective adult doses. For administration, ZQC granules and metformin were dissolved in distilled water immediately before use to prepare a homogeneous solution at the required concentration for oral gavage.

### 2.3 Serum sample preparation

Male Sprague–Dawley rats (6–8 weeks old, 200 ± 25 g) were randomly assigned to two groups (*n* = 5 per group) using a random number table: a normal control group and a ZQC group. The ZQC dose was calculated based on the human clinical equivalent dose of 4.14 g/kg and administered at 10 times this amount, according to classic methods for preparing drug-containing serum from traditional Chinese medicine (20). The control group received an equal volume of normal saline. Administration volume was 10 mL/kg, delivered twice daily for three consecutive days. One hour after the final administration, blood was collected from the abdominal aorta, and serum was separated by centrifugation. Serum was heat-inactivated at 56 °C for 30 min, filtered through a 0.22 μm membrane for sterilization, and stored at −80 °C until use.

### 2.4 Animal experiments

Male Lepr^−^/^−^ (db/db) mice (7 weeks, ~40 g), a widely used T2DM model that typically develops fatty liver and obesity, were employed to study diabetic MASLD (21–23). Age-matched Lepr^+^/^−^ (db/m, ~20 g) mice served as controls. After 1 week of acclimation, 8 db/m mice were assigned to the control group (CON), and 24 db/db mice were randomly divided into model (Mol), ZQC, and metformin (MET) groups (*n* = 8 each) using a random number table. Group allocation was concealed during sample collection and analysis.

ZQC (5.0 g/kg/day) and metformin (183 mg/kg/day) were administered by gavage; Mol received 0.9% NaCl. Treatments lasted 12 weeks, with weekly monitoring of body weight, food intake, and water consumption. At the end of the study, mice were anesthetized for cardiac blood collection. Plasma was stored at −80 °C, and liver and abdominal fat were harvested and weighed. The study was approved by the Animal Ethics Committee of Chengdu University of Traditional Chinese Medicine (Approval No.: 2024-236).

### 2.5 Construction of HG + PA HepG2 cell model

HepG2 cells were cultured in DMEM containing 5.5 mM D-glucose, 10% fetal bovine serum, and 1% penicillin–streptomycin at 37 °C in a 5% CO^2^ incubator. To simulate the metabolic environment of MASLD with T2DM, cells in the logarithmic growth phase were pretreated with DMEM containing 30 mM D-glucose for 24 h (HG), followed by 300 μM palmitic acid (PA) to induce intracellular lipid accumulation. Seven experimental groups were established: (1) Control (5.5 mM glucose); (2) Model (HG + PA); (3) HG + PA + 5%ZQC-containing serum (5% ZQC-S); (4) HG + PA + 7.5% ZQC-containing serum (7.5% ZQC-S); and (5) HG + PA + 7.5% ZQC-S + 10 μM GW1929 (PPARγ agonist, 2-h pretreatment).

### 2.6 Biochemical marker assay

Commercial biochemical assay kits (Shenzhen Mindray, China) were used to measure triglycerides (TG), total cholesterol(TC), high-density lipoprotein cholesterol (HDL-C), low-density lipoprotein cholesterol(LDL-C), alanine aminotransferase (ALT), and aspartate aminotransferase (AST) in mouse serum. All procedures were performed strictly according to the manufacturers’ instructions. Absorbance or concentration values were determined using an ELISA reader or a fully automated biochemical analyzer, and the concentrations of each marker were calculated based on standard curves.

### 2.7 Fasting blood glucose and insulin assay

Mice were fasted for 12 h each week, weighed, and blood was collected from the tail vein. Fasting blood glucose was measured using a glucose meter (EA-9, Sannuo Biosensor Co., Ltd., China). At the end of the experiment, serum insulin levels were determined using an ELISA kit (Wuhan E-L-R-E-T Biotechnology Co., Ltd., NO.E-EL-M1382c), and insulin resistance was calculated using the HOMA-IR formula: HOMA-IR = [fasting glucose (mmol/L) × fasting insulin (mU/L)]/22.5.

### 2.8 CCK-8 assay for cell viability

The CCK-8 assay was used to evaluate the effects of different concentrations of ZQC-containing serum on HepG2 cell viability. Cells were treated with 2.5, 5, 7.5, and 10%ZQC-containing serum and assessed at 24, 48, and 72 h post-treatment. Ten microliters of CCK-8 solution were added to each well, followed by incubation at 37 °C in the dark for 2 h. The optical density (OD) at 450 nm for each well was measured using a microplate reader.

### 2.9 Hematoxylin and eosin (H&E) and oil red O staining of liver tissue and HepG2 cells CCK-8 assay for cell viability

Paraffin-embedded liver sections were deparaffinized and rehydrated, and fixed HepG2 cell slides were prepared. Samples were first stained with H&E to visualize tissue and cellular morphology. For lipid detection, samples were immersed in 60% isopropanol and stained with fresh Oil Red O solution for 10–15 min under light protection, followed by hematoxylin counterstaining. All samples were mounted and imaged at 200× magnification to assess morphology and lipid droplet distribution.

### 2.10 Transmission electron microscopy (TEM) observation of liver ultrastructure

Mouse liver tissues were pre-fixed in 3% glutaraldehyde and post-fixed in 1% osmium tetroxide. Samples were dehydrated through graded acetone (30%–100%) and embedded in Epon-812 using stepwise ratios of dehydrating agent to resin (3:1, 1:1, 1:3). Ultrathin sections (60–90 nm) were mounted on copper grids and double-stained with uranyl acetate (10–15 min) followed by lead citrate (1–2 min). Ultrastructural images were acquired using a transmission electron microscope (JEM-1400FLASH, JEOL, Japan).

### 2.11 2-NBDG fluorescent staining for glucose uptake

HepG2 cells were seeded in well plates, starved in serum-free medium, and treated as indicated. Cells were then incubated with 100 μM 2-NBDG (from 1 mM stock) at 37 °C in the dark. After washing with PBS, fluorescence images were captured using a microscope (excitation 488 nm, emission 542 nm), and uptake was quantified with ImageJ software.

### 2.12 Gut microbiota 16S rRNA gene sequencing

Mouse fecal samples were collected and stored at −80 °C. Total microbial DNA was extracted using the Zymo BIOMICS DNA Microprep Kit. The V3–V4 region of the 16S rRNA gene was PCR-amplified using barcoded primers. Amplified products were verified by electrophoresis, and libraries were constructed using the NEBNext Ultra II DNA Library Prep Kit. Paired-end 250-bp sequencing was performed on the Illumina NovaSeq 6,000 platform. Raw data underwent quality control, assembly, and denoising to generate valid sequences for downstream analyses.

### 2.13 Non-targeted fecal metabolomics

Non-targeted metabolomics of gut microbial metabolites was performed on mouse fecal samples using GC–MS. Twenty milligrams of fecal material were extracted with methanol–water (4:1, v/v) containing an internal standard, followed by grinding, centrifugation, nitrogen drying, and methoxyamine N, E-oxime and BSTFA derivatization. GC–MS analysis was conducted on a DB-5MS column with helium as the carrier gas, programmed temperature ramping, and EI ionization in full-scan mode (m/z 50–500). Raw data were processed in MS-DIAL for peak detection, alignment, and matrix construction. Metabolites were annotated using the LuMet-GC 5.0 and NIST databases. Differential metabolites were screened in R software (VIP > 1, *p* < 0.05, FC ≥ 1.2 or ≤ 0.833), and pathway enrichment was conducted using KEGG. All analyses were completed on the Oebiotech cloud platform.

### 2.14 Liver transcriptome sequencing analysis

Total RNA was extracted from mouse liver tissues using a commercial RNA extraction kit and assessed for quality. Libraries were constructed following standard procedures, including rRNA depletion, RNA fragmentation, and strand-specific cDNA synthesis. Paired-end sequencing was performed on the Illumina NovaSeq 6,000 platform. Differentially expressed genes (DEGs) were identified using DESeq2 with thresholds of |log_2_FC| ≥ 2 and *p* < 0.01. Functional enrichment analysis of DEGs was performed using Gene Ontology (GO) and KEGG databases to systematically investigate potential signaling pathways.

### 2.15 Western blot analysis

Total protein was extracted from HepG2 cells using IP lysis buffer, followed by sonication and centrifugation. Protein concentration was determined by BCA assay and adjusted equally across samples. Proteins were mixed with 5 × loading buffer, denatured at 95 °C for 10 min, separated by SDS-PAGE, and transferred to membranes. Membranes were blocked with 5% nonfat milk in TBST for 2 h, incubated with primary antibodies overnight at 4 °C, and then with secondary antibodies for 2 h at room temperature. Bands were visualized using a Tanon chemiluminescence system, and intensities were quantified in ImageJ, normalizing target proteins to internal controls.

### 2.16 LC–MS analysis of ZQC

The chemical composition of ZQC was analyzed using LC–MS. Samples were extracted with methanol, centrifuged, and injected onto an ACQUITY Premier HSS T3 column with gradient elution in 0.1% formic acid–methanol. Mass spectrometry was performed using an ESI source in positive and negative ion modes. Full-scan resolution was set at 70,000 (m/z 150–1,500), with data-dependent MS^2^ acquisition of the top 10 ions at 17,500 resolution and collision energies of 10/30/55 eV. Data were processed in MS-DIAL and compounds were annotated using MassBank, ReSpect, and GNPS databases (MS1/MS2 tolerances 0.01/0.05 Da, identification threshold ≥80%).

### 2.17 Network pharmacology analysis of ZQC

The potential mechanisms of ZQC were investigated using network pharmacology. Targets of ZQC were predicted from its major LC–MS–identified components using the SwissTargetPrediction database. Disease-related targets for MASLD and T2DM were obtained from GeneCards, OMIM, and TTD. ZQC component targets were then intersected with these disease-related targets. A protein–protein interaction network was constructed using STRING and visualized in Cytoscape. The intersected targets were further analyzed for GO functional and KEGG pathway enrichment using DAVID, with *p* < 0.05 as the significance threshold.

### 2.18 Molecular docking analysis

Molecular docking was used to examine interactions between ZQC’s core active ingredients and key targets. Compound structures were obtained from PubChem, and protein structures from the PDB database. Receptors were preprocessed in PyMOL by removing water, separating ligands, and optimizing hydrogens. Docking was performed with AutoDock Vina 1.1.2, using known ligand positions to define binding sites. Binding strength was evaluated by free energy (ΔG, kcal/mol), and results were visualized in PyMOL.

### 2.19 Statistical analysis

Each experiment was performed in at least three independent replicates, with the results presented as mean ± standard error of the mean (SEM). Statistical analyses were then conducted using IBM SPSS Statistics 24 software. Specifically, two-group comparisons were made using unpaired Student’s t-tests, while for multiple-group comparisons, one-way analysis of variance (ANOVA) was performed. A *p*-value of <0.05 was considered statistically significant.

## 3 Results

### 3.1 ZQC improves glucose and lipid metabolism statistical analysis

**Figure 1:**
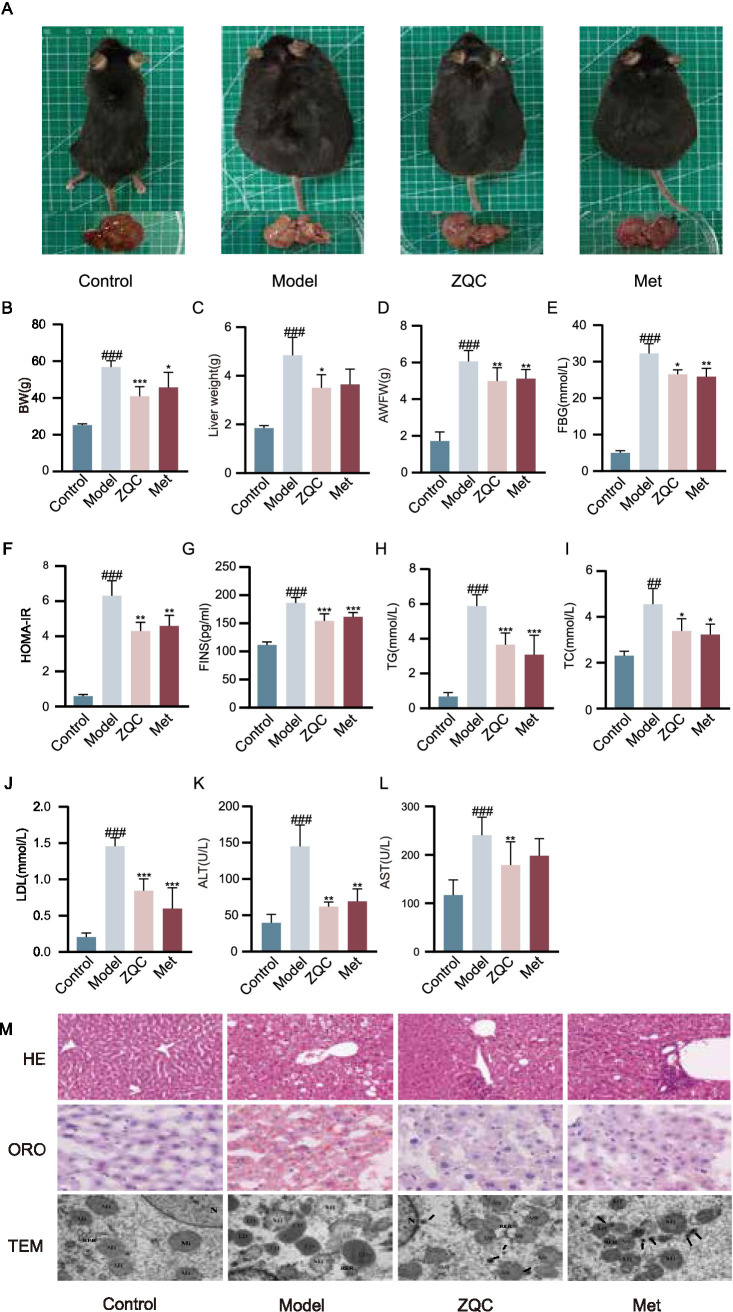
Effects of ZQC on glucose and lipid metabolism in db/db mice. **(A)** Representative images of mice and livers in the CON, model, ZQC, and Met groups. **(B–D)** BW, LW, and WAT weight in each group. **(E–G)** FBG, HOMA-IR, and FINS levels. **(H–L)** Serum TG, TC, LDL-C, ALT, and AST levels. **(M)** Representative liver images by H&E staining, Oil Red O staining, and TEM (H&E, 200×; Oil Red O, 400×). (^#^*p* < 0.05, ^##^*p* < 0.01, ^###^*p* < 0.001 vs. CON; ^*^p < 0.05, ^**^p < 0.01, ^***^p < 0.001 vs. Model).

Compared with the Control (CON) group, mice in the model (Mol) group showed marked increases in body weight, liver weight, and abdominal white adipose tissue (*p* < 0.001; Figures 1A–D). Treatment with ZQC or metformin effectively reversed these changes, significantly reducing these parameters (*p* < 0.05, *p* < 0.01, *p* < 0.001; Figures 1A–D). In terms of glucose metabolism, the model group showed markedly higher fasting blood glucose (FBG) and HOMA-IR index (*p* < 0.001; Figures 1E–G), accompanied by lower fasting insulin (FINS) levels, indicating pronounced insulin resistance. ZQC and metformin (Met) treatment significantly reversed these abnormalities (*p* < 0.05, *p* < 0.01, *p* < 0.001; Figures 1E–G). Furthermore, regarding lipid metabolism and liver function, serum triglycerides (TG), total cholesterol (TC), low-density lipoprotein cholesterol (LDL-C), and liver injury markers (ALT, AST) were markedly elevated in the model group (*p* < 0.01, *p* < 0.001; Figures 1H–L). Treatment with ZQC or metformin effectively mitigated these elevations (*p* < 0.05, *p* < 0.01, *p* < 0.001, Figures 1H–L).

### 3.2 ZQC attenuates hepatic steatosis and protects cellular ultrastructure

Histopathological examination of liver tissue further confirmed the therapeutic effects of ZQC. Oil Red O staining revealed that hepatocytes in the model group contained numerous red lipid droplets, indicating diffuse lipid accumulation, while ZQC and Met treatment markedly reduced lipid content (Figure 1M). H&E staining showed disorganized hepatocyte arrangement and extensive vacuolar steatosis in the model group. Treatment with ZQC or Met significantly decreased both vacuole number and size, restoring hepatocyte morphology toward a normal appearance (Figure 1M). Transmission electron microscopy revealed that hepatocytes in the model group exhibited swollen mitochondria with blurred or disrupted cristae and abundant lipid droplet accumulation. In contrast, hepatocytes from the ZQC and Met groups displayed well-defined mitochondrial structures, fewer lipid droplets, and numerous autophagosomes (Figure 1M). Together, these findings demonstrate that ZQC effectively reduces hepatic lipid accumulation and preserves normal hepatocyte ultrastructure.

### 3.3 ZQC modulates gut microbiota in T2DM with MASLD mice

**Figure 2:**
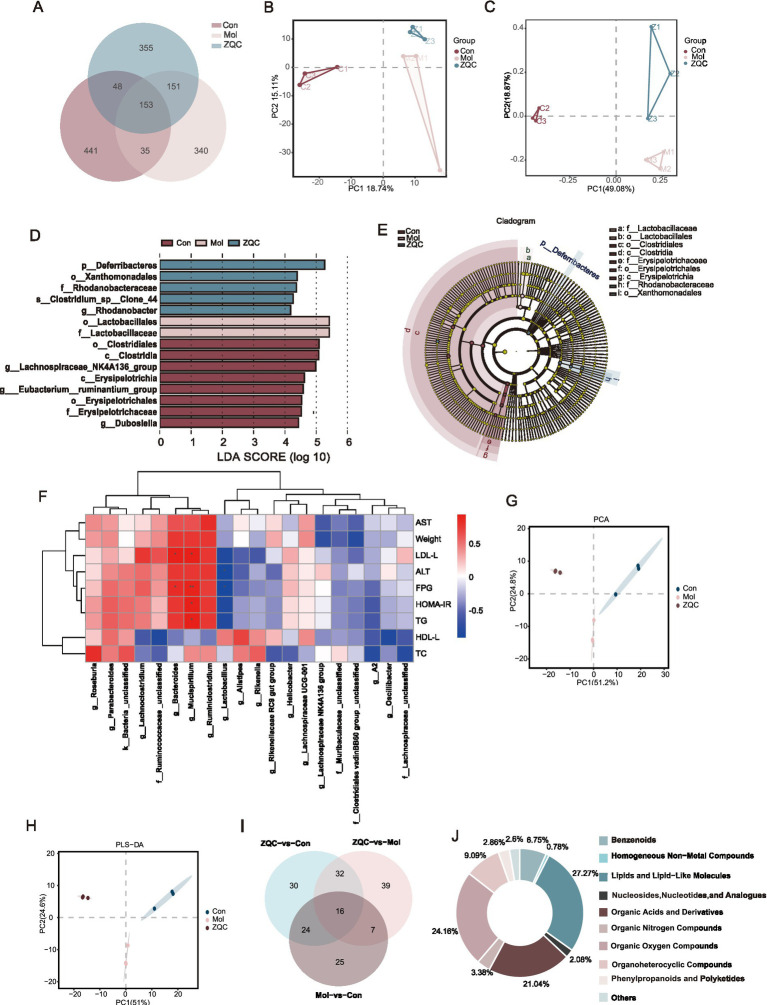
ZQC modulates gut microbiota composition and fecal metabolic profiles. **(A)** Venn diagram of fecal microbiota based on 16S RNA sequencing. **(B,C)** PCA and PCoA of gut microbiota among groups. **(D,E)** LDA score of differential taxa. **(F)** Correlation heatmap between genus-level gut microbiota (top 20) and metabolic parameters. **(G,H)** PCA and PLS-DA of fecal non-targeted metabolomics. **(I)** Venn diagram of differential fecal metabolites. **(J)** Proportional composition of fecal metabolites.

To systematically assess the effects of ZQC on gut microbiota, 16S rRNA sequencing was performed on mouse fecal samples. Across all samples, 1,523 amplicon sequence variants (ASVs) were identified, with 355 and 340 unique ASVs in the Mol and ZQC groups, respectively, indicating distinct microbial profiles among the groups (Figure 2A). Alpha diversity analysis, including Chao1, observed_species, and Shannon indices, revealed no significant differences among the groups (Supplementary Figure S1). However, beta diversity analysis using PCA and PCoA indicated that the gut microbiota of the model group deviated significantly, while ZQC treatment shifted it toward that of the control group, suggesting that ZQC can restore the overall structure of the disrupted microbial community (Figures 2B,C).

Furthermore, Linear Discriminant Analysis of Effect Size (LEfSe) was performed to identify taxa with significant differential abundance among groups (LDA threshold = 3). Results showed that the model group was primarily enriched in *Deferribacteres, Xanthomonadales, Rhodanobacteraceae*, and *Clostridium_sp Clone_44*, whereas the ZQC group was significantly enriched in *Lactobacillales* and *Lactobacillaceae*. Characteristic enriched taxa in the control group included *Lachnospiraceae NK4A13, Clostridiales, Clostridia, Erysipelotrichia, Dubosiella*, and *Eubacterium_ruminantium* (Figures 2D,E). Additionally, correlation analysis between genus-level gut microbiota and key metabolic parameters, including fasting plasma glucose (FPG), HOMA-IR, body weight, TC, TG, LDL-C, HDL-C, AST, and ALT, was performed using data from the model and ZQC groups. At the genus level, *Bacteroides* abundance was positively correlated with FPG and LDL-C, *Mucispirillum* abundance was positively correlated with FPG, HOMA-IR, and TG, while *Lactobacillus* abundance was negatively correlated with ALT and FPG (Figure 2F). These results indicate that specific gut microbial genera are closely associated with systemic metabolic and hepatic injury parameters, and suggest that ZQC-mediated microbial modulation is linked to improvements in glucose and lipid metabolism.

### 3.4 ZQC modulates gut metabolite profiles in T2DM with MASLD

**Figure 3:**
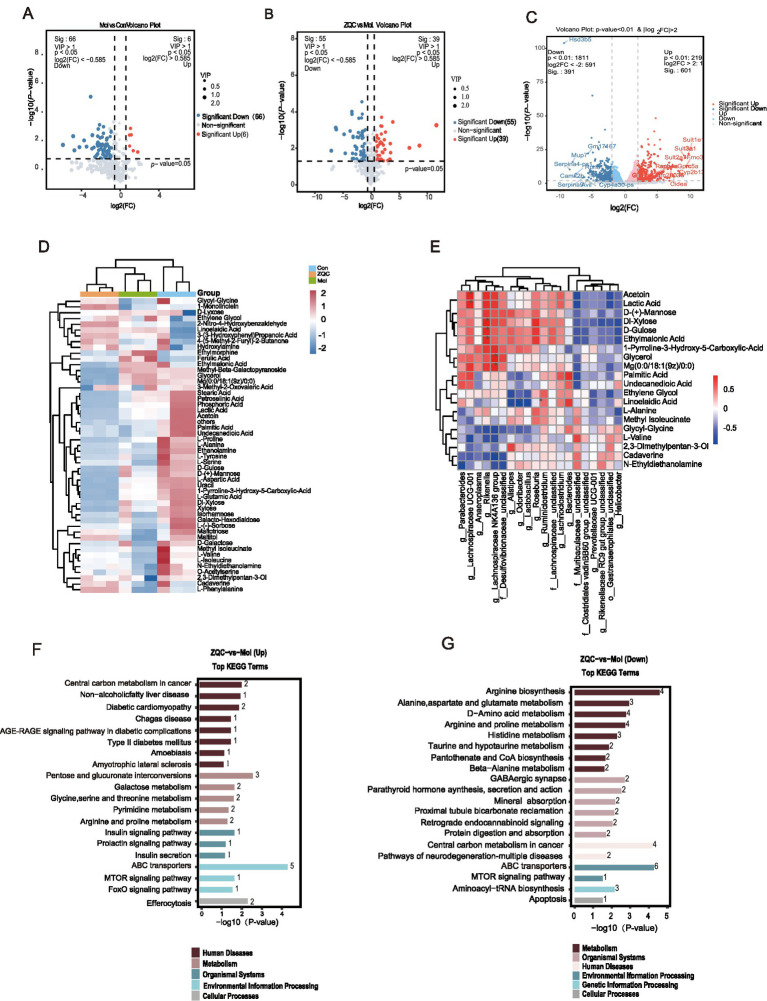
ZQC modulates fecal metabolome and liver transcriptome in db/db mice. **(A,B)** Volcano plots of differential fecal metabolites (Con vs. Mol; ZQC vs. Mol). **(C)** Volcano plots of differential hepatic RNAs (Con vs. Mol). **(D)** Heatmap of clustered differential metabolites. **(E)** Correlation heatmap between gut microbiota and differential metabolites. **(F,G)** KEGG enrichment of up and downregulated metabolites.

Non-targeted metabolomic analysis of mouse fecal samples showed clear separation among groups by PCA and PLS-DA, suggesting that ZQC effectively reversed disease-associated metabolic dysregulation (Figures 2G,H). A total of 385 metabolites were identified, mainly classified as lipids, organic acids, and oxygen-containing organic compounds (Figures 2I,J). Of these, 94 metabolites differed significantly between the Mol and ZQC groups (Figure 2I). Volcano plot analysis showed that, compared with the normal group, the model group displayed 6 upregulated and 66 downregulated metabolites (Figure 3A). In contrast, comparison between the model and ZQC groups revealed 39 upregulated and 55 downregulated metabolites in the model group (Figure 3B). These results indicate that ZQC partially reverses metabolic disturbances in T2DM with MASLD by modulating specific dysregulated metabolic pathways. Cluster analysis of these differential metabolites showed that maltitol, L-phenylalanine, and other metabolites were upregulated in the ZQC group, whereas ethylene glycol and others exhibited a decreasing trend (Figure 3D). These metabolites were enriched in several amino acid metabolic pathways, including arginine and proline metabolism, histidine metabolism, and glycine, serine, and threonine metabolism. Notably, metabolites upregulated by ZQC were significantly enriched in pathways closely associated with glucose and lipid metabolism, including non-alcoholic fatty liver disease (NAFLD), AGE-RAGE signaling, mTOR signaling, insulin secretion, type 2 diabetes, galactose metabolism, and pentose–glucuronic acid interconversion (Figures 3F,G).

Additionally, correlation analysis between gut microbiota at the genus level and differential metabolites revealed significant associations between specific bacterial taxa and host metabolites (Figure 3E). *Rikenella* was positively correlated with glycerol. The *Lachnospiraceae NK4A136* group showed a negative correlation with cadaverine and positive correlations with ethylmalonic acid, DL-xylose, and D-gulose (Figure 3E). *Lachnospiraceae UCG-001* was positively correlated with lactic acid and acetoin (Figure 3E). Linoelaidic acid exhibited positive correlations with both *Odoribacter* and *Lactobacillus*, while *Parabacteroides* was positively correlated with N-ethyldiethanolamine (Figure 3E). These results indicate close associations between ZQC-regulated gut microbiota and alterations in the fecal metabolite profile.

### 3.5 Hepatic transcriptomic profiling identifies key pathways modulated by ZQC in T2DM with MASLD

**Figure 4:**
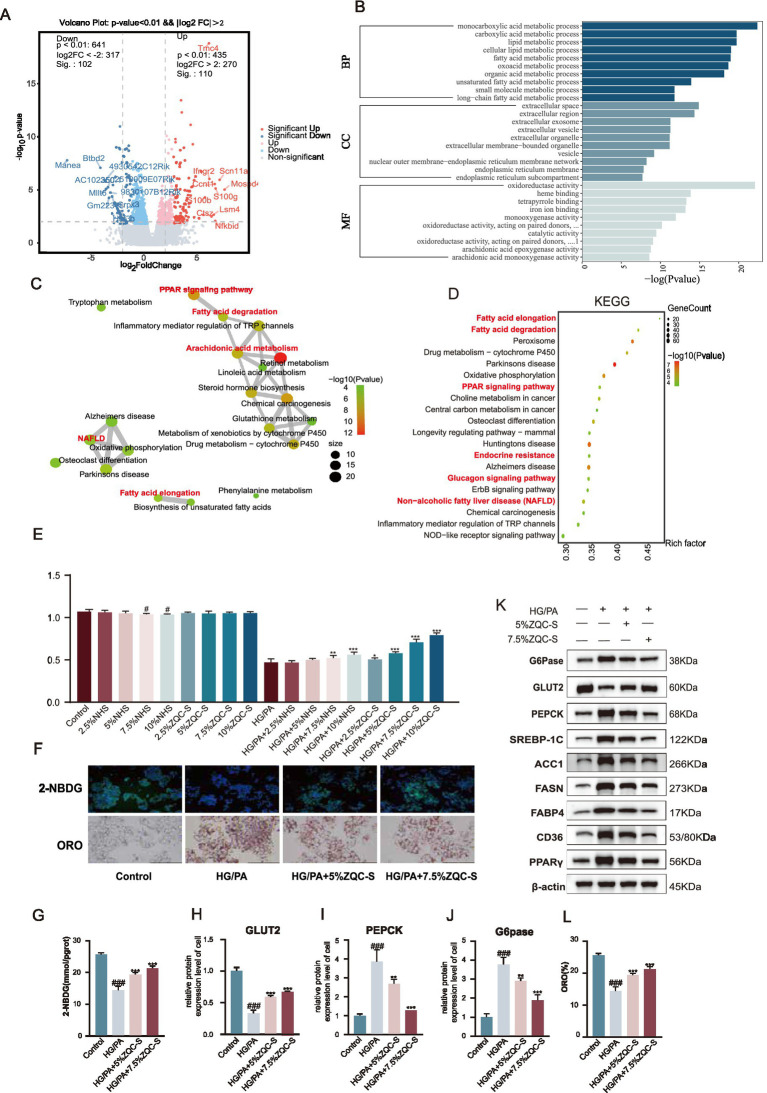
ZQC modulates glucose and lipid metabolism in HG/PA-induced HepG2 cells. **(A)** Volcano plots of differential hepatic RNAs (Mol vs. ZQC). **(B)** GO enrichment of differential RNAs in ZQC group. **(C,D)** KEGG enrichment of differential RNAs in ZQC group. **(E)** CCK8 assay showing cell viability at different serum concentrations. **(F)** 2-NBDG fluorescence and Oil Red O staining in Control, HG/PA, HG/PA + 5% ZQC-S, and HG/PA + 7.5% ZQC-S groups. **(G)** Quantification of 2-NBDG fluorescence intensity. **(H–J)** WB analysis of key gluconeogenic proteins (e.g., PEPCK, G6Pase, GLUT2). **(K)** Representative WB bands of selected glucose- and lipid-related proteins. **(L)** Quantification of lipid droplet content from Oil Red O staining. (^#^*p* < 0.05, ^##^*p* < 0.01, ^###^*p* < 0.001 vs. CON; ^*^p < 0.05, ^**^p < 0.01, ^***^p < 0.001 vs. HG/PA).

To further explore the molecular basis of ZQC intervention, RNA sequencing was performed on liver tissues. Compared with the control group, the model group exhibited 992 differentially expressed genes (DEGs), including 601 upregulated and 391 downregulated genes. In comparison, 212 DEGs were identified between the ZQC-treated group and the model group, comprising 110 upregulated and 102 downregulated genes (Figures 3H, 4A). GO enrichment analysis of DEGs in the ZQC group showed significant enrichment in lipid metabolism–related biological processes, including lipid metabolic process and fatty acid metabolic process. At the molecular function level, DEGs were enriched in arachidonic acid epoxygenase activity and arachidonic acid monooxygenase activity, while cellular component analysis indicated predominant localization in the extracellular region (Figure 4B). KEGG pathway analysis further revealed significant enrichment in the PPAR signaling pathway, along with multiple lipid metabolism–associated pathways, including arachidonic acid metabolism, fatty acid degradation, biosynthesis of unsaturated fatty acids, and NAFLD (Figures 4C,D).

### 3.6 ZQC improves glucose and lipid metabolism in HG/PA-induced hepatocytes

To investigate the effects of ZQC-containing serum on hepatocyte metabolic disorders, cell viability was first assessed to determine optimal concentrations. CCK-8 assay results showed that, compared with the HG + PA model group, ZQC-containing serum at concentrations of 2.5%–10% significantly increased cell survival (*p* < 0.05, *p* < 0.001), with the most pronounced effect observed at 5%–7.5% (*p* < 0.001). This concentration range was therefore used for subsequent experiments (Figure 4E).

Glucose metabolism was evaluated using the 2-NBDG fluorescent probe. The HG + PA model significantly impaired hepatic glucose uptake, whereas ZQC treatment effectively restored this capacity (Figures 4F,G). Western blot analysis further demonstrated that ZQC treatment downregulated the expression of key gluconeogenic enzymes PEPCK and G6Pase, while upregulating the glucose transporter GLUT2 (*p* < 0.01, *p* < 0.001; Figures 4H,K).

**Figure 5:**
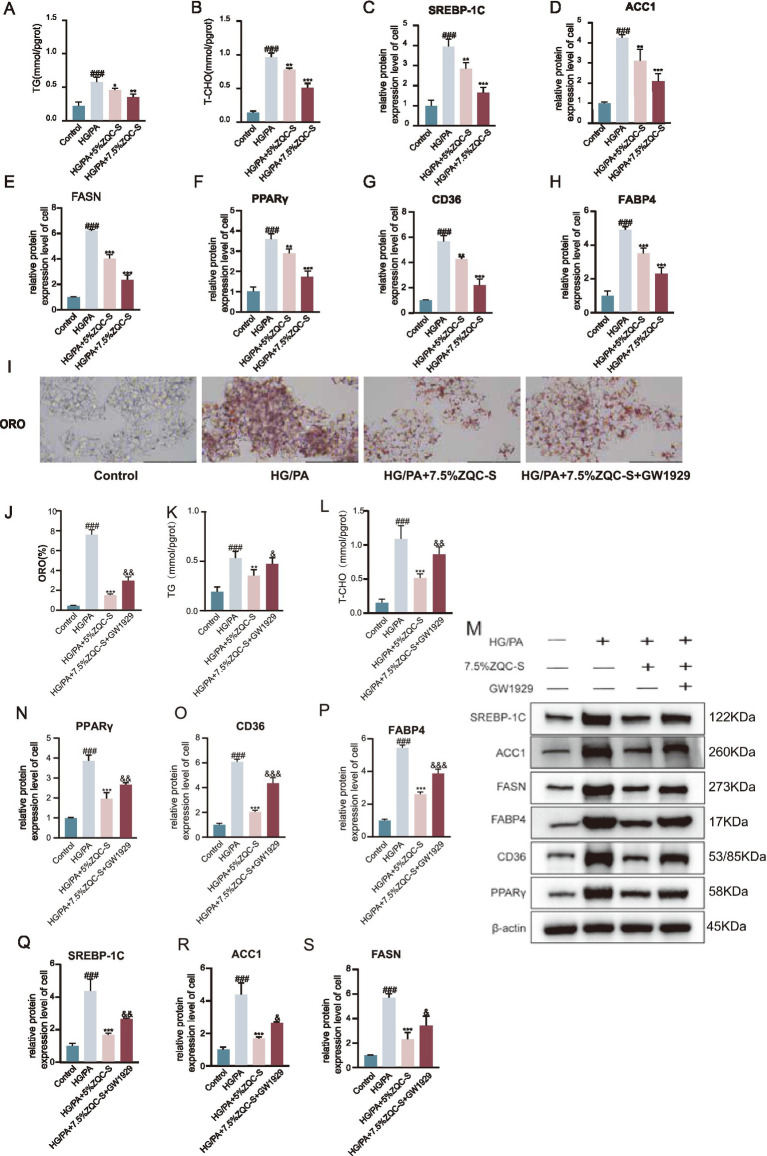
ZQC regulates lipid metabolism via the PPARγ pathway in HG/PA-induced HepG2 cells. **(A,B)** Intracellular TG and T-CHO levels. **(C,D)** Relative expression of proteins (e.g., SREBP-1c, ACC1). **(E–H)** WB quantification of representative lipogenic and PPARγ pathway proteins (FASN, PPARγ, CD36, FABP4) in control, HG/PA, HG/PA + 5% ZQC-S, and HG/PA + 7.5% ZQC-S groups. **(I)** Representative Oil Red O staining images of lipid droplets. **(J)** Quantification of lipid droplet content (ORO%) in each group. **(K,L)** Intracellular TG and T-CHO levels. **(M)** WB analysis of key proteins in control, HG/PA, HG/PA + 7.5% ZQC-S, and HG/PA + 7.5% ZQC-S + GW1929 groups. **(N–S)** Relative expression of selected lipid metabolism and PPARγ pathway proteins across the four groups. (^#^*p* < 0.05, ^##^*p* < 0.01, ^###^*p* < 0.001 vs. CON; *p < 0.05, **p < 0.01, ***p < 0.001 vs. HG/PA; ^&^*p* < 0.05, ^&&^*p* < 0.01, ^&&&^*p* < 0.001 vs. HG/PA + 5% ZQC-S + g1929).

In terms of lipid metabolism, Oil Red O staining revealed substantial accumulation of lipid droplets in HG + PA-treated hepatocytes, with some droplets forming clusters that extensively covered the cytoplasm. ZQC treatment significantly reduced both the number and size of lipid droplets, resulting in markedly diminished staining intensity compared with the HG + PA model (*p* < 0.001; Figures 4F,L). Biochemical analysis further confirmed that intracellular triglyceride (TG) and total cholesterol (T-CHO) levels were significantly elevated in the HG + PA model group compared with the control (*p* < 0.001), and ZQC treatment markedly attenuated these increases (*p* < 0.05, *p* < 0.01, *p* < 0.001; Figures 5A,B).

### 3.7 ZQC improves lipid metabolism by modulating the PPARγ pathway and its functional validation

To investigate the molecular mechanisms by which ZQC alleviates lipid accumulation, we examined the expression of key proteins involved in fatty acid synthesis and the PPARγ signaling pathway. Western blot analysis showed that HG + PA treatment significantly upregulated the expression of SREBP-1c, a central transcription factor in fatty acid synthesis, along with its downstream targets ACC1 and FASN (*p* < 0.001, Figures 5C–E). In addition, HG + PA treatment markedly increased the expression of PPARγ and its downstream fatty acid transporters CD36 and FABP4 (*p* < 0.001, Figures 5B–D). ZQC treatment effectively downregulated all these proteins, with the most pronounced effect observed at 7.5% ZQC concentration (*p* < 0.01, *p* < 0.001; Figures 5F–H).

After determining 7.5% as the optimal concentration for ZQC-containing serum, the PPARγ agonist GW1929 was introduced for co-intervention experiments to further investigate the role of the PPARγ pathway in ZQC-mediated regulation of lipid metabolism. Oil Red O staining showed that, compared with the HG + PA + 7.5% ZQC group, co-treatment with GW1929 markedly exacerbated lipid accumulation in HepG2 cells (*p* < 0.01; Figures 5I,J). Consistently, biochemical assays indicated significant increases in total cholesterol (T-CHO) and triglyceride (TG) levels (*p* < 0.05, *p* < 0.01; Figures 5K,L). Western blot analysis demonstrated that under HG + PA-induced conditions, the expression of PPARγ and its downstream targets CD36 and FABP4, as well as fatty acid synthesis-related proteins SREBP-1c, ACC1, and FASN, was significantly elevated in HepG2 cells (*p* < 0.001; Figures 5M–S). Intervention with ZQC-containing serum markedly reduced the expression of these proteins (*p* < 0.001; Figures 5M–S). Notably, co-treatment with GW1929 significantly upregulated PPARγ, CD36, and FABP4 (*p* < 0.01, *p* < 0.001; Figures 5M–P), accompanied by a renewed increase in SREBP-1c, ACC1, and FASN expression (*p* < 0.05, *p* < 0.01; Figures 5M,Q–S).

### 3.8 ZQC compound identification and network pharmacology

**Figure 6:**
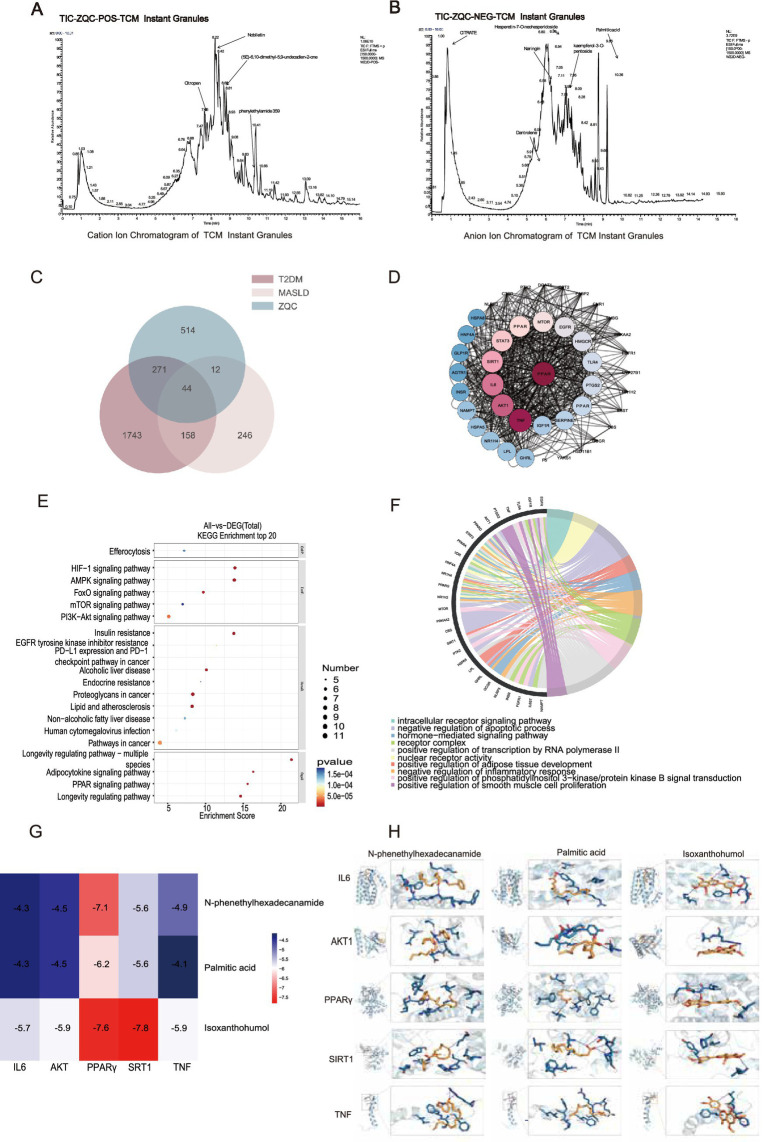
LC–MS and network pharmacology analysis of ZQC. **(A,B)** LC–MS chromatograms of ZQC in positive and negative ion modes. **(C)** Venn diagram of overlapping targets between ZQC components and MASLD/T2DM. **(D)** PPI network of ZQC for MASLD with T2DM. **(E)** GO enrichment analysis (top 10). **(F)** KEGG enrichment analysis (top 20). **(G)** Heatmap of binding energies between key ZQC components and targets (red: strong binding, blue: weak binding). **(H)** Molecular docking of key ZQC components with targets.

LC–MS analysis of ZQC generated the total ion current chromatogram (Figures 6A,B). A total of 19 major chemical constituents were identified in ZQC through screening. These compounds mainly belong to the flavonoid, organic acid/fatty acid, terpenoid, and steroid classes. The top five most abundant compounds were Nobiletin, citrate, N-phenylethylamide, (5E)-6,10-dimethyl-5,9-undecadien-2-one, and Citropen (Supplementary Table S1).

Network pharmacology analysis identified 44 common targets shared by ZQC active components and MASLD/T2DM (Figure 6C). Based on the PPI network constructed in Cytoscape 3.9.0, the top key targets ranked by degree values included PPARγ, TNF, AKT1, IL6, and SIRT1 (Figure 6D). Construction of the target–compound network identified the top five core chemical components based on degree values, including Isoxanthohumol, palmitic acid, and N-phenethylhexadecanamide (Supplementary Table S2). These compounds are likely to represent the core mechanisms through which ZQC modulates T2DM with MASLD.

GO and KEGG enrichment analyses using DAVID identified the top 10 GO terms and top 20 KEGG pathways. Notably enriched pathways comprised the PPAR signaling pathway, inflammatory response, adipose tissue development, hormone-mediated signaling, insulin resistance, lipid and atherosclerosis, and non-alcoholic fatty liver disease (Figures 6E,F).

### 3.9 Molecular docking

Semi-rigid molecular docking was performed using AutoDock to evaluate interactions between the top three key compounds (N-phenethylhexadecanamide, Palmitic acid, Isoxanthohumol) and the top five key targets (PPARγ, TNF, AKT1, IL6, SIRT1) (Figure 6H). Among the 15 protein–compound pairs, nine exhibited binding affinities below −5.0 kcal/mol, indicating stable interactions (Figure 6G). These results suggest that ZQC compounds may modulate these targets to exert hypoglycemic and lipid-lowering effects.

## 4 Discussion

Type 2 diabetes mellitus (T2DM) complicated by metabolic-associated steatotic liver disease (MASLD) is a complex metabolic comorbidity. It is characterized by disruption of the gut-liver axis, insulin resistance, and dysregulated lipid metabolism, yet the underlying molecular mechanisms remain incompletely understood (24–27). The liver is the central regulator of systemic glucose and lipid metabolism, and its dysfunction plays a key role in disease progression. Abnormal lipid metabolism, enhanced triglyceride synthesis, and impaired very low-density lipoprotein (VLDL) secretion lead to excessive hepatic lipid accumulation (28, 29). This hepatic lipid accumulation further aggravates insulin resistance, increases hepatic glucose output, and places additional stress on pancreatic *β*-cells, thereby driving a vicious cycle of glucose and lipid dysregulation (30, 31). Chronic hyperglycemia accelerates hepatic lipid deposition and hepatocellular injury, promoting progression toward NASH and liver fibrosis (3, 4). Our results show that ZQC markedly reduces hepatic lipid burden, alleviates steatosis and hepatocellular injury, and improves glucose and lipid homeostasis.

Accumulating evidence indicates that the gut microbiota plays a central role in metabolic diseases such as obesity, fatty liver disease, and T2DM (5). Through the portal vein, the liver continuously receives microbial cells and their metabolites from the gut, thereby directly modulating hepatic lipid metabolism, insulin sensitivity, and inflammatory responses (32). In this study, ZQC improved glucose and lipid metabolism by reshaping the disrupted gut microbiota in model mice. Although *α*-diversity indices (Chao1 and Shannon) showed no significant changes, this does not imply that ZQC lacked efficacy, as metabolic benefits are often mediated primarily through alterations in microbial composition and function rather than overall diversity. LEfSe analysis revealed significant enrichment of the *Lactobacillales* order and *Lactobacillaceae* family following ZQC intervention. Consistently, correlation analysis demonstrated a significant negative association between *Lactobacillus* abundance and both fasting plasma glucose (FPG) and alanine aminotransferase (ALT) levels, suggesting a potential contribution of *Lactobacillus* to ZQC-mediated improvements in glycemic control and liver function. *Lactobacillales*, recognized probiotics, strengthen the intestinal barrier and limit systemic endotoxin (LPS) absorption, alleviating hepatic inflammation, while also improving glucose and lipid metabolism via modulation of insulin signaling and promotion of insulin secretion (33–35). Concurrently, ZQC also increased the abundance of *Lachnospiraceae* and *Muribaculaceae*, which are known SCFA-producing taxa that contribute to intestinal redox homeostasis and support optimal gut-liver signaling (36).

At the genus level, *Bacteroides* abundance was positively correlated with FPG and LDL-C levels, whereas *Mucispirillum* showed significant positive correlations with FPG, HOMA-IR, and triglyceride levels. Previous studies have reported that *Mucispirillum* preferentially thrives in high-fat environments, with its abundance progressively increasing during disease progression in high-fat/high-cholesterol diet–induced NAFLD models, and is closely associated with hepatic steatosis, steatohepatitis, and hepatocellular carcinoma development (37, 38). *Bacteroidetes*, key probiotics for carbohydrate fermentation, produce short-chain fatty acids (SCFAs) that activate GPR41 and GPR43 receptors to stimulate gut-derived insulinotropic hormone GLP-1 secretion, thereby enhancing *β*-cell function and insulin sensitivity (33, 34, 39, 40). Once transported to the liver, these SCFAs can further modulate lipid metabolism–related genes, including PPARγ, ultimately contributing to reduced hepatic lipid droplet accumulation (41). Collectively, these findings indicate that remodeling of the gut microbiota represents a key mechanism through which ZQC exerts its beneficial effects on hepatic metabolic homeostasis.

Non-targeted metabolomic analysis further elucidates the mechanisms by which ZQC mediates its effects via the “microbiota–metabolite-host signaling” axis. ZQC induces the reconfiguration of multiple pathways closely associated with hepatic metabolism, including the metabolism of amino acids such as arginine, proline, histidine, glycine, serine, and threonine; the pentose-glucuronic acid interconversion pathway; and networks related to AGE-RAGE and mTORC1. As a central hub for amino acid metabolism, the liver is highly responsive to fluctuations in these metabolites, which serve not only as energy substrates but also modulate lipid oxidation, gluconeogenesis, and inflammatory signaling (42–46). Notably, glycine has been shown to suppress lipid synthesis, enhance fatty acid oxidation, and mitigate inflammation, consistent with the metabolic benefits observed in this study (47). Conversely, aberrant activation of the AGE-RAGE axis enhances hepatic stellate cell activation, stimulates pro-inflammatory cytokine secretion, and impairs insulin signaling, further exacerbating metabolic stress and hepatocellular injury (47). Correlation analysis between gut microbiota and metabolites further revealed that the *Lachnospiraceae NK4A136* group and *Rikenella* displayed significant positive correlations with multiple metabolites, suggesting that these taxa may indirectly influence hepatic glucose and lipid homeostasis via their metabolic products. Liver transcriptomic analysis demonstrated that ZQC intervention substantially modulated pathways related to lipid metabolism, including fatty acid metabolism, cellular lipid metabolic processes, and arachidonic acid metabolism. These pathways collectively encompass fatty acid oxidation, insulin signaling, inflammatory responses, and fibrosis regulation, supporting ZQC’s multi-targeted modulation of hepatic metabolism (48–50). Together, these pathways regulate fatty acid oxidation, insulin signaling, inflammation, and fibrosis, supporting ZQC’s multi-targeted effects on liver metabolism.

Liver transcriptome analysis revealed significant enrichment of the PPAR signaling pathway, and network pharmacology analysis identified PPARγ as the core target of ZQC in T2DM with MASLD, showing the highest degree value and strongest binding affinity to its main active component. PPARγ is a central transcription factor regulating adipocyte differentiation, lipid metabolism, and insulin sensitivity, exhibiting bidirectional regulatory properties (51, 52). It serves as a well-established target for insulin sensitization, and agonists such as TZDs can effectively improve glucose metabolism (53). However, overexpression or aberrant activation of PPARγ in hepatocytes is a hallmark of fatty liver disease, whereas its expression is low in healthy human livers (54). Studies indicate that under metabolic stress, excessive activation of hepatic PPARγ synergistically upregulates fatty acid transporters, including CD36, and lipid synthases, such as ACC and FAS, promoting fatty acid uptake and triglyceride synthesis, thereby exacerbating hepatic lipid accumulation and insulin resistance (54). The findings in our HG + PA-induced hepatocyte model closely mirror these pathological mechanisms. ZQC did not activate the PPARγ pathway; instead, it restrained its pathological overactivation under metabolic stress, as evidenced by the downregulation of PPARγ and its downstream proteins involved in fatty acid transport and synthesis. Co-treatment with the PPARγ-specific agonist GW1929 partially reversed these effects, confirming that ZQC’s metabolic benefits depend on modulating this pathway. Overall, these results indicate that ZQC targets hepatic PPARγ overactivation to fine-tune lipid uptake and synthesis, providing a novel mechanistic insight into the improvement of glucose and lipid dysregulation in T2DM with MASLD.

Unlike many traditional Chinese medicine formulas that primarily exert metabolic benefits through direct activation of PPARγ signaling (55, 56), ZQC appears to function as a context-dependent modulator rather than a classical agonist. Given the tissue and condition specific roles of PPARγ, restraining its aberrant hepatic activation under metabolic stress may be more favorable for alleviating steatosis while preserving insulin sensitivity. This regulatory mode distinguishes ZQC mechanistically from conventional PPARγ-activating compounds and may contribute to its balanced improvement of glucose and lipid metabolism.

Studies have shown that traditional Chinese herbal medicines exhibit promising efficacy in treating T2DM with MASLD through multiple mechanisms, including glucose-lowering effects, lipid regulation, and modulation of gut microbiota (57, 58). LC–MS analysis, network pharmacology, and molecular docking collectively demonstrated that the key active components of ZQC (phenylethylamide, palmitic acid, and isoxanthohumol) can directly interact with PPARγ, SIRT1, AKT1, and associated signaling pathways, thereby modulating the production and functional effects of gut microbiota-derived metabolites. These findings provide a molecular basis linking ZQC’s chemical composition to gut microbiota–associated metabolic regulation. Among these components, isoxanthohumol has been reported to improve dysregulated glucose and lipid metabolism (59), modulate gut microbiota composition (60) and related metabolites (61), and inhibit intestinal lipid absorption (62), suggesting that it may contribute to the metabolic effects of ZQC.

In summary, this study preliminarily establishes a multidimensional regulatory network linking the chemical composition of ZQC, gut microbiota, microbial metabolites, PPARγ signaling, and hepatic metabolic regulation. This integrated framework provides a mechanistic basis for understanding the systemic therapeutic effects of ZQC and offers a foundation for its further development and potential clinical translation.

## 5 Conclusion

This study demonstrated for the first time that Zhaqu Compound (ZQC) exerts protective effects on T2DM with MASLD *in vitro* and *in vivo*. ZQC improved glucose and lipid metabolism, reshaped gut microbiota, and modulated hepatic PPARγ signaling by restraining its pathological overactivation. Isoxanthohumol, one of the key active components, may play a central role in these effects. Overall, our findings indicate that ZQC has multi-target therapeutic potential for T2DM with MASLD and provide a mechanistic basis for its further development.

## 6 Limitations and perspectives

Several limitations of this study should be noted. First, the regulatory role of ZQC on the PPARγ pathway was primarily validated at the cellular level, and direct in vivo verification in animal models remains limited. Although transcriptomic analysis, network pharmacology, and pharmacological intervention collectively support the involvement of PPARγ signaling, future studies incorporating animal-level validation, such as hepatic protein expression analysis or pathway-specific modulation, are needed to further confirm causality. Second, only a single therapeutic dose of ZQC was evaluated in this study, which restricts assessment of dose–response relationships and optimal intervention ranges. Given the multicomponent and multitarget nature of ZQC, systematic multi-dose investigations integrating metabolic, microbiota, and signaling outcomes will be essential. Addressing these limitations will further strengthen the mechanistic understanding and translational potential of ZQC in the treatment of T2DM with MASLD.

## Data Availability

The datasets presented in this study can be found in online repositories. The names of the repository/repositories and accession number(s) can be found at: https://ngdc.cncb.ac.cn/omix/edit/OMIX013929, OMIX013929; https://ngdc.cncb.ac.cn/gsub/submit/bioproject/subPRO080079/overview, PRJCA054487; https://ngdc.cncb.ac.cn/gsub/submit/bioproject/subPRO079845/overview, PRJCA054344.
