# Supplementary material for: Zhaqu compound improves glucose and lipid metabolism in T2DM with MASLD by modulating gut microbiota and PPARγ
Source: Front Nutr. 2026 Mar 27;13:1775686. doi: 10.3389/fnut.2026.1775686 (PMC13067941; doi:10.3389/fnut.2026.1775686)
Supplement: Supplementary file 1 [file Table_1.DOCX]

Supplementary Material

## Supplementary Figures


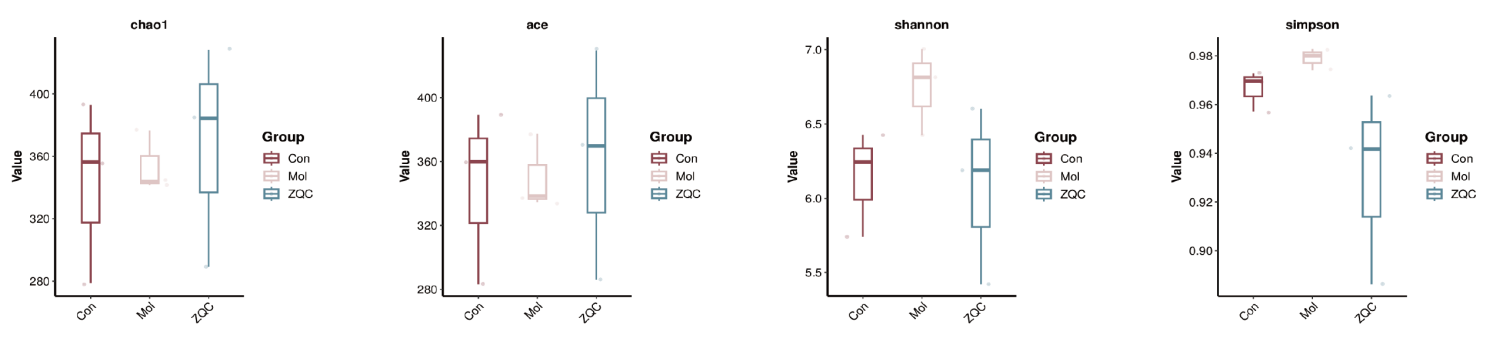


Supplementary Fig. 1 1. Rank-Abundance Curve of Gut Microbiota α-Diversity


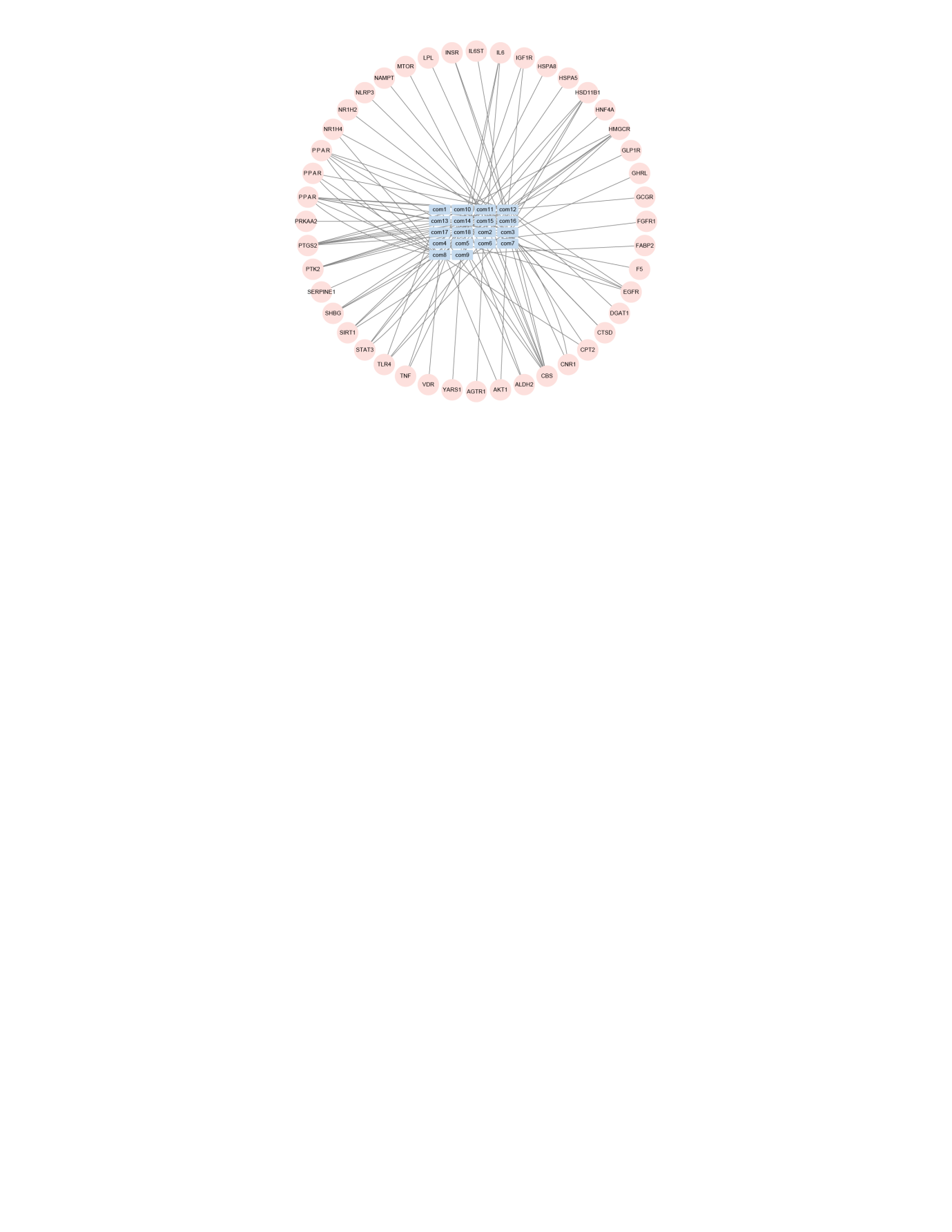


Supplementary Fig. 2 Component-target network(com1: Nobiletin; com2: CITRATE; com3: phenylethylamide 359; com4: (5E)-6,10-dimethyl-5,9-undecadien-2-one; com5: Citropen; com6: Naringin; com7: Dantrolene; com8: Palmitic acid; com9: Hesperetin-7-O-neohesperidoside; com10: kaempferol-3-O-pentoside; com11: Isoxanthohumol; com12: Isosinensetin; com13: FA 18:1+3O; com14: Cryptotanshinone; com15: zapotin; com16: (1R,2E,7R,10E,12S,13S,15R)-12,15-dihydroxy-7-methyl-8-oxabicyclo[11.3.0]hexadeca-2,10-dien-9-one; com17: Hesperidin; com18: D-(+)-Trehalose)

1. Supplementary Tables

2.1Supplementary Tab. 1. Analysis of the Main Chemical Constituents of ZQC

| **Title** | **RT**  **(min)** | **Precursor m/z** | **Adduct** | **Reference m/z** | **Formula** | **Ontology** | **Total score** | | **Ratio%** |
| --- | --- | --- | --- | --- | --- | --- | --- | --- | --- |
| Nobiletin | 8.235217 | 403.1385 | [M+H]+ | 403.13873 | C_21_H_22_O_8_ | 8-O-methylated flavonoids | 87.6 | 5.301022907 | |
| CITRATE | 1.062033 | 191.0191 | [M-H]- | 191.01973 | C_6_H_8_O_7_ | Tricarboxylic acids and derivatives | 96.8 | 5.254985602 | |
| phenylethylamide 359 | 10.41442 | 360.3251 | [M+H]+ | 360.32608 | C_24_H_41_NO | N-acyl amines | 99.6 | 3.069558546 | |
| (5E)-6,10-dimethyl-5,9-undecadien-2-one | 8.701 | 217.1596 | [M+H]+ | 217.16 | C_13_H_22_O | Acyclic monoterpenoids | 99.9 | 2.852940905 | |
| Citropen | 7.674233 | 207.0658 | [M+H]+ | 207.06599 | C_11_H_10_O_4_ | Coumarins and derivatives | 85.8 | 2.820467408 | |
| Naringin | 6.584367 | 579.1721 | [M-H]- | 579.17194 | C_27_H_32_O_14_ | Flavonoid-7-O-glycosides | 93 | 2.238393477 | |
| Dantrolene | 5.786083 | 313.0568 | [M-H]- | 313.05783 | C_14_H_10_N_4_O_5_ | Hydantoins | 99.5 | 2.052061036 | |
| Palmitic acid | 9.859433 | 255.233 | [M-H]- | 255.23296 | C_16_H_32_O_2_ | Long-chain fatty acids | 83.9 | 1.934080501 | |
| Hesperetin-7-O-neohesperidoside | 6.877234 | 609.183 | [M-H]- | 609.1825 | C_28_H_34_O_15_ | Flavonoid-7-O-glycosides | 88.8 | 1.746393681 | |
| kaempferol-3-O-pentoside | 7.300317 | 417.0833 | [M-H]- | 417.08273 | C_20_H_18_O_10_ | Flavonoid-3-O-glycosides | 99.9 | 1.649310338 | |
| Isoxanthohumol | 7.802617 | 355.1521 | [M+H]+ | 355.14999 | C_21_H_22_O_5_ | 8-prenylated flavanones | 78.5 | 1.635503433 | |
| Isosinensetin | 7.984066 | 373.1296 | [M+H]+ | 373.13 | C_20_H_20_O_7_ | 8-O-methylated flavonoids | 87 | 1.597860455 | |
| FA 18:1+3O | 7.973883 | 329.2339 | [M-H]- | 329.23334 | C_18_H_34_O_5_ | Long-chain fatty acids | 70.9 | 1.319718921 | |
| Cryptotanshinone | 8.831133 | 297.1499 | [M+H]+ | 297.14853 | C_19_H_20_O_3_ | Tanshinones, isotanshinones, and derivatives | 87.8 | 1.282882365 | |
| zapotin | 8.164166 | 343.1187 | [M+H]+ | 343.11761 | C_19_H_18_O_6_ | 6-O-methylated flavonoids | 78 | 1.279443611 | |
| (1R,2E,7R,10E,12S,13S,15R)-12,15-dihydroxy-7-methyl-8-oxabicyclo[11.3.0]hexadeca-2,10-dien-9-one | 8.831133 | 319.1312 | [M+H]+ | 319.13062 | C_16_H_24_O_4_ | Macrolides and analogues | 99.8 | 1.171617178 | |
| Hesperidin | 6.709883 | 609.1828 | [M-H]- | 609.1825 | C_28_H_34_O_15_ | Flavonoid-7-O-glycosides | 88.1 | 1.150151301 | |
| D-(+)-Trehalose | 1.535067 | 341.1101 | [M-H]- | 341.10895 | C_12_H_22_O_11_ | O-glycosyl compounds | 93.1 | 1.074684149 | |

2.2 Supplementary Tab. S2. Degree information for PPI

| **NO.** | **name** | **Degree** |
| --- | --- | --- |
| 1 | PPARG | 68 |
| 2 | TNF | 66 |
| 3 | AKT1 | 60 |
| 4 | IL6 | 58 |
| 5 | SIRT1 | 54 |
| 6 | STAT3 | 50 |
| 7 | PPARA | 48 |
| 8 | MTOR | 46 |
| 9 | EGFR | 42 |
| 10 | HMGCR | 40 |
| 11 | PTGS2 | 38 |
| 12 | TLR4 | 38 |
| 13 | PPARD | 36 |
| 14 | SERPINE1 | 34 |
| 15 | IGF1R | 32 |
| 16 | GHRL | 30 |
| 17 | NAMPT | 30 |
| 18 | HSPA5 | 30 |
| 19 | NR1H4 | 30 |
| 20 | LPL | 30 |
| 21 | INSR | 24 |
| 22 | AGTR1 | 22 |
| 23 | HNF4A | 22 |
| 24 | GLP1R | 22 |
| 25 | HSPA8 | 20 |
| 26 | CTSD | 18 |
| 27 | NLRP3 | 18 |
| 28 | PTK2 | 16 |
| 29 | CPT2 | 16 |
| 30 | DGAT1 | 16 |
| 31 | CNR1 | 14 |
| 32 | FABP2 | 14 |
| 33 | FGFR1 | 12 |
| 34 | SHBG | 12 |
| 35 | PRKAA2 | 12 |
| 36 | CYP27B1 | 10 |
| 37 | IL6ST | 10 |
| 38 | NR1H2 | 10 |
| 39 | GCGR | 8 |
| 40 | CBS | 8 |
| 41 | HSD11B1 | 6 |
| 42 | YARS1 | 4 |
| 43 | F5 | 2 |

2.3 Supplementary Table S3 lists the top 50 enriched terms of biological processes, cellular components, and molecular functions identified by GO analysis of the ZQC network pharmacology targets.

| **Category** | **GO** | **Description** | **q-value** | **Enrichment_score** |
| --- | --- | --- | --- | --- |
| molecular_function | GO:0046965 | nuclear retinoid X receptor binding | 0.000248522422204361 | 91.5176470588235 |
| molecular_function | GO:0051117 | ATPase binding | 0.0493458602733646 | 9.97307692307692 |
| biological_process | GO:0070374 | positive regulation of ERK1 and ERK2 cascade | 0.0146524291407367 | 9.84683544303797 |
| molecular_function | GO:0140297 | DNA-binding transcription factor binding | 0.0146524291407367 | 9.84683544303797 |
| biological_process | GO:0006954 | inflammatory response | 0.000917782894407364 | 9.6037037037037 |
| biological_process | GO:0043123 | positive regulation of canonical NF-kappaB signal transduction | 0.0058769472090335 | 9.58004926108374 |
| biological_process | GO:0050727 | regulation of inflammatory response | 0.0496872225865219 | 9.48658536585366 |
| biological_process | GO:0045766 | positive regulation of angiogenesis | 0.0424468428225303 | 9.41008064516129 |
| molecular_function | GO:0000976 | transcription cis-regulatory region binding | 0.0063934680627198 | 9.34975961538461 |
| cellular_component | GO:0043202 | lysosomal lumen | 0.0496872225865219 | 9.26071428571429 |
| biological_process | GO:0010628 | positive regulation of gene expression | 0.000522949947931587 | 9.23627968337731 |
| molecular_function | GO:0019899 | enzyme binding | 0.00115171558153829 | 9.12492668621701 |
| molecular_function | GO:0030296 | protein tyrosine kinase activator activity | 0.00187618290834364 | 89.7576923076923 |
| biological_process | GO:0060396 | growth hormone receptor signaling pathway | 0.0063934680627198 | 86.4333333333333 |
| biological_process | GO:0072540 | T-helper 17 cell lineage commitment | 0.00668267290509116 | 86.4333333333333 |
| biological_process | GO:0009755 | hormone-mediated signaling pathway | 0.000110424796246124 | 80.4724137931034 |
| biological_process | GO:0008283 | cell population proliferation | 0.0424468428225303 | 8.83977272727273 |
| biological_process | GO:0010629 | negative regulation of gene expression | 0.0069167528723989 | 8.72085201793722 |
| cellular_component | GO:0045121 | membrane raft | 0.0174325814428138 | 8.59558011049724 |
| biological_process | GO:0001525 | angiogenesis | 0.00813628251282939 | 8.27553191489362 |
| biological_process | GO:0046697 | decidualization | 0.00041513547688558 | 70.7181818181818 |
| biological_process | GO:0010763 | positive regulation of fibroblast migration | 0.00876436696732228 | 70.7181818181818 |
| biological_process | GO:0010884 | positive regulation of lipid storage | 0.00876436696732228 | 70.7181818181818 |
| biological_process | GO:0014912 | negative regulation of smooth muscle cell migration | 0.00876436696732228 | 70.7181818181818 |
| molecular_function | GO:0031994 | insulin-like growth factor I binding | 0.00876436696732228 | 70.7181818181818 |
| biological_process | GO:0046209 | nitric oxide metabolic process | 0.00898844121904328 | 70.7181818181818 |
| biological_process | GO:0071377 | cellular response to glucagon stimulus | 0.00944786991159713 | 70.7181818181818 |
| biological_process | GO:0140052 | cellular response to oxidised low-density lipoprotein particle stimulus | 0.00944786991159713 | 70.7181818181818 |
| biological_process | GO:0008284 | positive regulation of cell population proliferation | 0.002695315949619 | 7.87746835443038 |
| biological_process | GO:0030154 | cell differentiation | 0.00115171558153829 | 7.79632516703786 |
| biological_process | GO:0006629 | lipid metabolic process | 0.0424468428225303 | 7.52806451612903 |
| molecular_function | GO:0061629 | RNA polymerase II-specific DNA-binding transcription factor binding | 0.0424468428225303 | 7.47980769230769 |
| cellular_component | GO:0098794 | postsynapse | 0.0424468428225303 | 7.02921686746988 |
| biological_process | GO:0046889 | positive regulation of lipid biosynthetic process | 0.00304112182828127 | 68.6382352941176 |
| biological_process | GO:1905564 | positive regulation of vascular endothelial cell proliferation | 0.00304112182828127 | 68.6382352941176 |
| molecular_function | GO:0004879 | nuclear receptor activity | 0.000110424796246124 | 67.6434782608696 |
| molecular_function | GO:0003707 | nuclear steroid receptor activity | 0.00306235438785142 | 61.4131578947368 |
| molecular_function | GO:0043565 | sequence-specific DNA binding | 0.0313182282703232 | 6.88407079646018 |
| molecular_function | GO:0005102 | signaling receptor binding | 0.0160654618950159 | 6.63737201365188 |
| molecular_function | GO:0001228 | DNA-binding transcription activator activity, RNA polymerase II-specific | 0.0069167528723989 | 6.53697478991597 |
| molecular_function | GO:0003700 | DNA-binding transcription factor activity | 0.00401492276670305 | 6.52913669064748 |
| cellular_component | GO:0010008 | endosome membrane | 0.041916709878532 | 6.45560165975104 |
| biological_process | GO:0045892 | negative regulation of DNA-templated transcription | 0.00355727350349633 | 6.35020408163265 |
| cellular_component | GO:0043025 | neuronal cell body | 0.0174325814428138 | 6.31412337662338 |
| biological_process | GO:0007166 | cell surface receptor signaling pathway | 0.0439257157900469 | 6.27338709677419 |
| biological_process | GO:0045944 | positive regulation of transcription by RNA polymerase II | 0.000134225784024478 | 6.04194174757282 |
| biological_process | GO:0015909 | long-chain fatty acid transport | 0.0113797812511403 | 59.8384615384615 |
| biological_process | GO:0038083 | peptidyl-tyrosine autophosphorylation | 0.0113797812511403 | 59.8384615384615 |
| molecular_function | GO:0043560 | insulin receptor substrate binding | 0.0119515171897488 | 59.8384615384615 |
| biological_process | GO:0045348 | positive regulation of MHC class II biosynthetic process | 0.0119515171897488 | 59.8384615384615 |

2.4 Supplementary Table S4 lists the top 20 enriched terms of biological processes, cellular components, and molecular functions identified by KEGG analysis of the ZQC network pharmacology targets.

| **NO.** | **Category** | **Description** |  | **LogP** | **Enrichment** | **ListHits** |
| --- | --- | --- | --- | --- | --- | --- |
| 1 | KEGG Pathway | AMPK signaling pathway |  | -8 | 14 | 9 |
| 2 | KEGG Pathway | Longevity regulating pathway - multiple species |  | -8 | 15 | 7 |
| 3 | KEGG Pathway | HIF-1 signaling pathway |  | -7 | 16 | 8 |
| 4 | KEGG Pathway | Insulin resistance |  | -7 | 17 | 8 |
| 5 | KEGG Pathway | Longevity regulating pathway |  | -6 | 18 | 7 |
| 6 | KEGG Pathway | Proteoglycans in cancer |  | -6 | 19 | 9 |
| 7 | KEGG Pathway | Lipid and atherosclerosis |  | -6 | 20 | 9 |
| 8 | KEGG Pathway | Adipocytokine signaling pathway |  | -6 | 21 | 6 |
| 9 | KEGG Pathway | PPAR signaling pathway |  | -6 | 22 | 6 |
| 10 | KEGG Pathway | Alcoholic liver disease |  | -5 | 23 | 7 |
| 11 | KEGG Pathway | FoxO signaling pathway |  | -5 | 24 | 7 |
| 12 | KEGG Pathway | PI3K-Akt signaling pathway |  | -4 | 25 | 9 |
| 13 | KEGG Pathway | Pathways in cancer |  | -4 | 26 | 11 |
| 14 | KEGG Pathway | EGFR tyrosine kinase inhibitor resistance |  | -4 | 27 | 5 |
| 15 | KEGG Pathway | PD-L1 expression and PD-1 checkpoint pathway in cancer |  | -4 | 28 | 5 |
| 16 | KEGG Pathway | Human cytomegalovirus infection |  | -4 | 29 | 7 |
| 17 | KEGG Pathway | Non-alcoholic fatty liver disease |  | -4 | 30 | 6 |
| 18 | KEGG Pathway | Efferocytosis |  | -4 | 31 | 6 |
| 19 | KEGG Pathway | Endocrine resistance |  | -4 | 32 | 5 |
| 20 | KEGG Pathway | mTOR signaling pathway |  | -4 | 33 | 6 |
